# Effectiveness and Safety of Omidenepag Isopropyl 0.002% Ophthalmic Solution in Treatment-Naive Patients With Primary Open Angle Glaucoma: A Prospective Multicenter Phase IV Study

**DOI:** 10.1097/IJG.0000000000002605

**Published:** 2025-06-17

**Authors:** Hyoung Won Bae, Eun Ji Lee, Jong Jin Jung, Ki Ho Park

**Affiliations:** * Yonsei University Severance Hospital; † Bundang Seoul National University Hospital; ‡ Kim’s Eye Hospital; § Seoul National University Hospital, Seoul, Korea

**Keywords:** primary open angle glaucoma, omidenepag isopropyl 0.002% ophthalmic solution, EP2 receptor agonist, intraocular pressure, phase IV study

## Abstract

**Précis:**

Omidenepag isopropyl is a selective E-prostanoid subtype 2 (EP2) receptor agonist that lowers intraocular pressure. Omidenepag isopropyl 0.002% ophthalmic solution is effective and safe to use at the first diagnosis of primary open angle glaucoma.

**Purpose:**

To evaluate the effectiveness and safety of omidenepag isopropyl 0.002% ophthalmic solution in treatment-naive patients at first diagnosis of primary open angle glaucoma (POAG) in real-world clinical settings in Korea.

**Patients and Methods:**

In a single-arm, multicenter, open-label, prospective, phase IV clinical trial, patients with newly diagnosed POAG received omidenepag isopropyl 0.002% (one drop once daily) for 12 weeks. The primary endpoint was the change from baseline in intraocular pressure (IOP) at week 12. Secondary endpoints included change from baseline in IOP at week 4; change from baseline in IOP at week 12 in a subgroup with normal tension glaucoma (NTG); occurrences, incidence rates and changes from baseline in safety-related indicators (macular edema, endothelial cell count, central corneal thickness, prostaglandin-associated periorbitopathy syndrome). Safety was assessed by the occurrence of adverse events (AEs).

**Results:**

The effectiveness analysis set comprised 37 patients and the safety analysis set 50 patients. Mean IOP decreased from 16.19±2.65 mm Hg at baseline to 13.55±2.46 mm Hg at week 12 (*P*<0.0001), representing a 16% reduction. Mean reduction in IOP was 15% at week 4 (*P*<0.0001); and 16% at week 12 (*P*<0.0001) in the NTG subgroup (n=31). Aside from conjunctival injection, no notable changes were observed in safety-related evaluation indicators. The most common AEs were hyperemia (13 cases) and iridocyclitis (5 cases). No systemic AEs were reported.

**Conclusion:**

Omidenepag isopropyl 0.002% ophthalmic solution is suitable for first-line use at first diagnosis of POAG, including in patients with NTG.

Glaucoma is a collection of eye disorders characterized by progressive deterioration of the optic nerve, which can lead to irreversible vision loss and blindness.^1^ Primary open angle glaucoma (POAG) is the most common form of glaucoma accounting for 3-quarters of all cases.^2^ The global population with POAG was around 80 million in 2020 and the numbers are expected to rise rapidly as the population ages.^3,4^

In POAG, the anterior chamber angle is open (unobstructed) and appears normal, but aqueous humor outflow is diminished. Although elevated intraocular pressure (IOP) is a common feature of POAG, the condition can occur without elevated IOP, often referred to as normal tension glaucoma (NTG). Since ocular hypertension is the only modifiable causative factor for glaucoma, treatment is aimed at lowering IOP to slow disease progression.^5^

Topical medications, particularly prostaglandin F receptor agonizts (latanoprost and others), are the usual first-line choice to treat POAG. FP agonizts effectively reduce IOP by enhancing the uveoscleral outflow of aqueous humor and offer a convenient once-daily dosing schedule.^6^ However, not all patients with POAG respond to FP agonizts and these agents have the potential to cause adverse ocular effects. Along with common side effects such as conjunctival hyperemia and eye irritation, chronic treatment with FP agonizts can cause distinctive local effects, including deepening of the upper eyelid sulcus (DUES), pigmentation of the iris and periorbital skin, uneven thickening and elongation of eyelashes, and cystoid macular edema.^6^ As such, efforts have been made to identify novel therapeutics with a more favorable safety profile.

Omidenepag isopropyl is the first selective E-prostanoid subtype 2 (EP2) receptor agonist to exhibit IOP-lowering activity. Omidenepag isopropyl lowers and controls IOP by increasing aqueous humor drainage through both the trabecular meshwork and uveoscleral outflow pathways.^7^ As omidenepag isopropyl provides stable 24-hour IOP reduction,^8^ it is suitable for once-daily administration.^9^ Importantly, omidenepag isopropyl has less propensity than FP agonizts to induce prostaglandin-associated peri-orbitopathy syndrome (PAPS).^10,11^

An extensive clinical development program supported the regulatory approval of omidenepag isopropyl 0.002% ophthalmic solution for treating POAG and ocular hypertension. In phase 3 clinical trials, omidenepag isopropyl 0.002% ophthalmic solution was shown to be effective and well tolerated in non/low responders to latanoprost;^12,13^; was noninferior to latanoprost 0.005%^14,15^ and timolol 0.5%;^16^ and demonstrated sustained IOP reduction and acceptable safety over 52 weeks of use.^16,17^ A common feature of these phase 3 studies was that relatively few patients had no previous exposure to IOP-lowering agents. Japanese investigators found that new administration of omidenepag isopropyl 0.002% ophthalmic solution in untreated patients with POAG significantly reduced IOP, suggesting its suitability for first-line use.^18^

The current study aimed to evaluate the effectiveness and safety of omidenepag isopropyl 0.002% ophthalmic solution in treatment-naive patients at first diagnosis of POAG in real-world clinical settings as evidence of benefit could expand treatment options for this patient group.

## METHODS

### Study Design

This was a single-arm, multicenter, open-label, prospective phase IV clinical trial conducted at 4 ophthalmic centers in Seoul, Korea: Seoul National University Hospital, Yonsei University Severance Hospital, Bundang Seoul National University Hospital and Kim Ophthalmology Hospital. Patients with newly diagnosed POAG who met the inclusion/exclusion criteria and agreed to participate in the study were enrolled sequentially. Following approval of the study protocol by the Institutional Review Board of each institution, the study was conducted in accordance with the ethical principles of the Declaration of Helsinki and Korean Good Clinical Practice (KGCP) and related regulations. All patients provided written consent to participate in the clinical trial.

### Inclusion/Exclusion Criteria

Eligible patients were adults aged 19–80 years; newly diagnosed with POAG; with confirmed glaucomatous visual field defects through a visual field test within 6 months, defined as an open angle on gonioscopy [anterior chamber angle Grade ≥2 (Shaffer scale) in both eyes], retinal nerve fiber layer defects, or glaucomatous optical disc changes (neuroretinal rim thinning, disc excavation, or disc hemorrhage); were not being treated with glaucoma medications; and had a baseline IOP of 10–34 mm Hg. NTG was defined as a baseline IOP of ≤ 21 mm Hg.

Exclusion criteria: glaucoma due to secondary causes such as pseudoexfoliative glaucoma or pigment dispersion syndrome; severe visual field depression (mean deviation ≤−20 dB and/or visual field index ≤40); refractive abnormalities (outside of ± 6.00 D in sphere or 2.00 D in cylinder); a history of eye surgery within 6 months of the date of consent; a history of corneal reflexive surgery, including LASIK, LASEK, etc.; corneal abnormalities that may interfere with the Goldmann application tonometry test; severe dry eyes (taking medication or needing medication), active external eye disease, or an eye inflammatory disease, such as iris inflammation or uveitis; macular edema, retinal detachment, diabetic retinopathy, or retinal disease at risk of exacerbation; blood pressure ≥140/90 mm Hg; a history of injuries around the eyes, surgical history, or thyroid orbitopathy that may affect evaluation of PAPS; use of systemic or ophthalmic steroids; women who were pregnant, lactating, or planning to become pregnant within 6 months of consent; need for contact lenses during the clinical trial period; pseudophakia or aphakia; a history of hypersensitivity to investigational drugs; those deemed unfit to participate in the clinical trial by investigators.

### Trial Procedures

The study period was 12 weeks. Patients underwent procedure-specific tests at screening, baseline, week 4 (±7 d), and week 12 (±7 d). Eye tests included mean ocular perfusion pressure, anterior segment photography, visual acuity test, refraction test, IOP test, slit-lamp biomicroscopy, goniscopy, fundus photography, stereoscopic optic disc photography, standard automated perimetry, IOLMaster (axial length and central corneal thickness), spectral-domain optical coherence tomography for retinal nerve fiber layer thickness and specular microscope. All tests for effectiveness evaluations were implemented according to standardized test methods.

Patients applied one drop of omidenepag isopropyl 0.002% ophthalmic solution to the affected eye(s) each evening at 9 PM (±1 h). Use of artificial tears was permitted for patients with dry eyes. Patients were given ophthalmic diaries to record the number of drops of study medication and dry eye medication (as required). Medication compliance was assessed at 4 and 12 weeks.

### Primary Endpoint

The primary endpoint was the change from baseline in IOP at week 12. IOP was measured by Goldmann applanation tonometry. If the difference between 2 measurements was <2 mm Hg, the average value was used. If the difference between 2 measurements was ≥ 2 mm Hg, a third measurement was taken and the median value was used. IOP was measured at 10 AM±1 hour.

### Secondary Endpoints

Secondary endpoints were: change from baseline in IOP at week 4; change from baseline in IOP at week 12 in a subgroup with NTG; reaction rate of IOP at week 12 (proportion of patients with IOP lowered by more than 20% compared with baseline); number of occurrences, incidence rates, and changes in safety-related evaluation indicators (conjunctival hyperemia, macular edema, endothelial cell count, central corneal thickness, and PAPS) during the clinical trial period.

Safety was assessed by the occurrence of adverse events (AEs) during the observation period. AEs were coded according to system organ class and preferred term using the Medical Dictionary for Regulatory Activities (MedDRA). The number of patients who experienced coded AEs, the incidence rate, and the number of occurrences were recorded.

### Statistical Analysis

With reference to outcomes recorded for previously untreated patients with POAG in the AYAME study,^14^ 45 patients were required to perform a paired *t* test with a 5% significance level and 90% power. Assuming a dropout rate of 10% during 12 weeks’ observation, the target sample size was set at 50 patients.

The effectiveness analysis group included all patients who received the study drug, excluding those who stopped treatment, dropped out of the trial or had serious violations of inclusion and exclusion criteria. A subgroup analysis was planned, excluding patients whose drug compliance was <70%. Missing data were not replaced for patients who dropped out or were not followed up.

Summary statistics are used: mean and SD for continuous variables; and absolute frequency (n) and relative frequency (%) for categorical variables.

For the primary endpoint (change from baseline in IOP at week 12), the mean and SD are presented. The mean difference between baseline and week 12 was compared using a paired *t* test. If normality was not satisfied with the Shapiro-Wilk or Kolmogorov-Smirnov test, changes were compared using the Wilcoxon signed-rank test. The same statistical procedure was used for the secondary endpoints of change from baseline in IOP at week 4 and change from baseline in IOP at week 12 in the NTG subgroup.

All analyses were performed using SAS Ver.9.4 (SAS Institute, NC) software.

## RESULTS

The study was conducted between 19 April 2022 and 17 July 2023.

**FIGURE 1:**
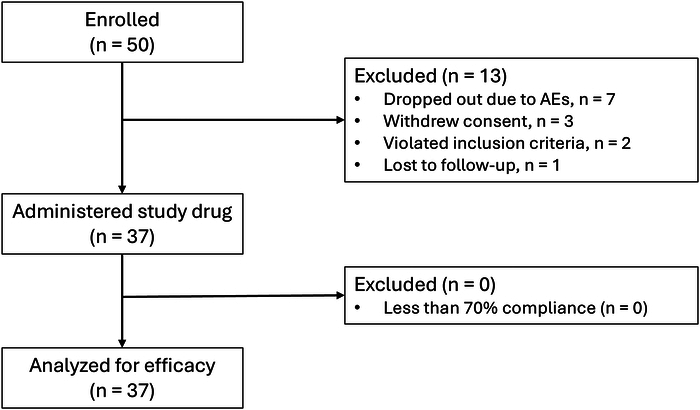
Patient disposition. AEs, adverse events.

Among 50 patients enrolled, 13 were excluded from the effectiveness analysis set. Thirty-seven patients (25 male), all with ≥70% medication compliance, met the criteria for the effectiveness analysis (Fig. 1). All 50 enrolled patients were analyzed for safety.

Mean age in the effectiveness analysis set was 49.7±12.1 years. The mean IOP at baseline was 16.19±2.65 mm Hg.

### Primary Endpoint

**FIGURE 2:**
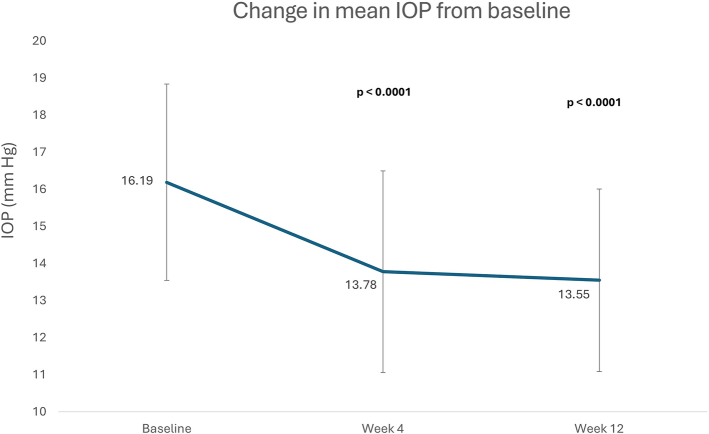
Primary endpoint: change in mean intraocular pressure (IOP) from baseline to week 12.

The mean IOP was 16.19±2.65 mm Hg at baseline and 13.55±2.46 mm Hg at week 12. The mean change in IOP of −2.64±2.09 mm Hg from baseline to week 12 was statistically significant (*P*<0.0001) (Fig. 2).

### Secondary Endpoints

The mean IOP was 16.19±2.65 mm Hg at baseline and 13.78±2.72 mm Hg at week 4. The mean change in IOP of −2.41±2.24 mm Hg from baseline to week 4 was statistically significant (*P*<0.0001) (Fig. 2).

**FIGURE 3:**
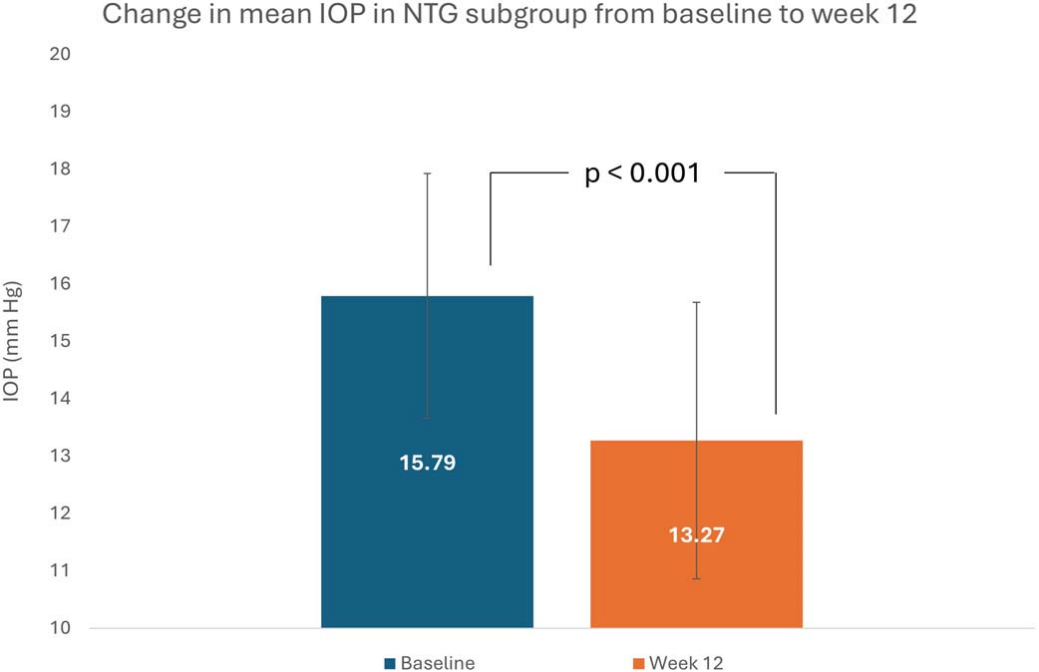
Change in mean intraocular pressure (IOP) from baseline to week 12 in the patient subgroup (n=31) with normal tension glaucoma (NTG).

In the NTG subgroup (n=31), the mean IOP was 15.79±2.13 mm  Hg at baseline and 13.27±2.41 mm Hg at week 12. The mean change in IOP of −2.52±2.12 mm Hg from baseline to week 12 was statistically significant (*P*<0.0001) (Fig. 3).

**FIGURE 4:**
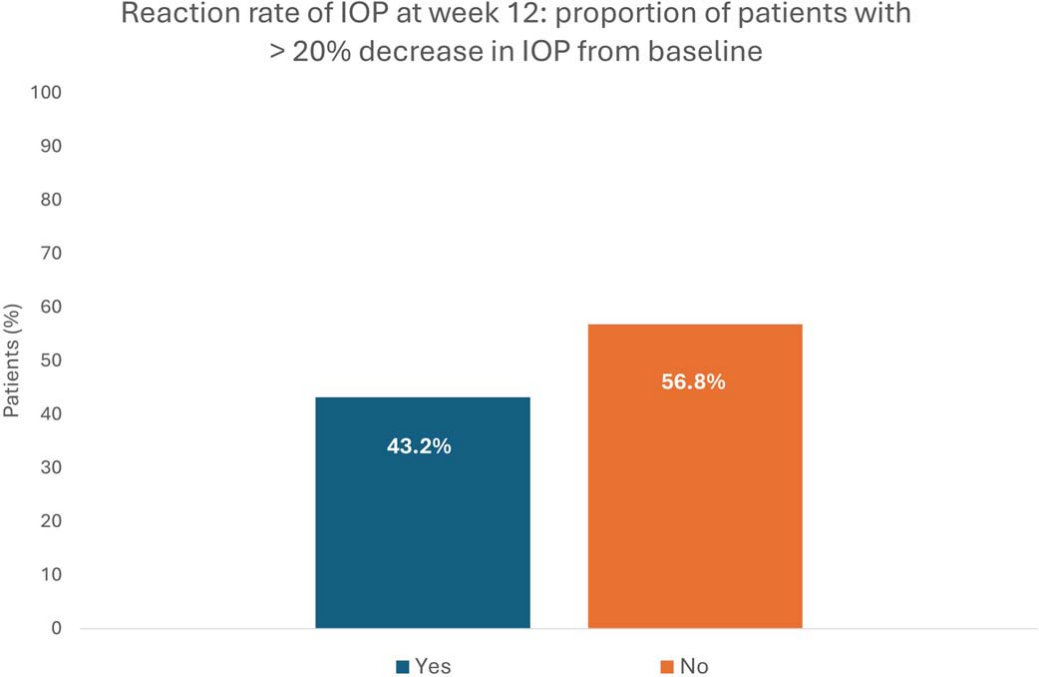
Reaction rate of intraocular pressure (IOP): proportion of patients with a >20% reduction in IOP at week 12.

Sixteen patients (43.2%) recorded a decrease in IOP of more than 20% from baseline to week 12, for an incidence rate of 0.43 (95% CI: 0.27–0.59) (Fig. 4).

The number of occurrences, incidence rates, and changes in safety-related evaluation indicators during 12 weeks’ observation are summarized in Table 1. The incidence rate of conjunctival injection increased from baseline (0.03; 95% CI: 0.00–0.08) to week 4 (0.62; 95% CI: 0.47–0.78), then decreased to week 12 (0.24; 95% CI: 0.12–0.38). Incidence rates of macular edema were 0.00 (0 cases) at baseline, weeks 4 and 12. There were no notable changes in mean values for corneal endothelial cell count parameters or central corneal thickness from baseline to week 12. There were no cases of PAPS at baseline (incidence rate of 0.00). Two cases of DUES were observed at week 4 (0.05; 95% CI: 0.00–0.13) and week 12 (0.05; 95% CI: 0.00–0.13) in the same patients. Both cases were reported as slight or mild in severity.

**TABLE 1:** Number of Occurrences, Incidence Rates, and Changes in Safety-Related Evaluation Indicators in Patients With Primary Open Angle Glaucoma During Treatment With Omidenepag Isopropyl 0.002% Ophthalmic Solution

	Patients (n=37)			
Parameter	Timepoint	Incidence cases	Incidence rate	95% CI
Conjunctival injection	Baseline	1	0.03	(0.00– 0.08)
	Week 4	23	0.62	(0.47– 0.78)
	Week 12	9	0.24	(0.12– 0.38)
Macular edema	Baseline	0	0.00	—
	Week 4	0	0.00	—
	Week 12	0	0.00	—
Corneal endothelial cell count				
CD, mm^2^	Baseline	Mean 2770.03	SD 215.90	(2698.04– 2842.01)
	Week 12	Mean 2786.39	SD 212.78	(2714.39– 2858.38)
CV	Baseline	Mean 33.14	SD 5.62	(31.26– 35.01)
	Week 12	Mean 34.92	SD 7.29	(32.45– 37.38)
HEX	Baseline	Mean 51.46	SD 8.35	(48.68– 54.24)
	Week 12	Mean 49.33	SD 8.16	(46.57– 52.10)
NUM	Baseline	Mean 192.24	SD 40.58	(178.71– 205.77)
	Week 12	Mean 182.56	SD 49.62	(165.77– 199.34)
Central corneal thickness, μm	Baseline	Mean 549.00	SD 31.09	(538.63– 559.37)
	Week 4	Mean 565.58	SD 30.74	(555.18– 575.99)
Prostaglandin-associated perio-orbitopathy syndrome	Baseline	0	0.00	—
	Week 4	2	0.05	(0.00– 0.13)
	Week 12	2	0.05	(0.00– 0.13)

CD indicates cell density; CV, coefficient of variation; HEX, hexagonality; NUM, number of cells (excluding inaccurate count cells).

Twenty-three patients (46.0%) experienced a total of 38 AEs, of which 36 AEs (94.8%) were judged as “mild” and required no specific action to be taken (Table 2). The most common AEs reported were hyperemia (13 cases) and iridocyclitis (5 cases). At study end, 35 (92.1%) of the AEs had “recovered/resolved.” With respect to causality, 31 (81.6%) of the AEs were considered possibly (76.3%) or probably/likely (5.3%) related to treatment.

**TABLE 2:** Adverse Events Reported in Patients With Primary Open Angle Glaucoma During Treatment With Omidenepag Isopropyl 0.002% Ophthalmic Solution

	Patients (n=50), n (%)
Overall incidence	23 (46.0)
Incidence by preferred term	
Hyperemia	13 (34.2)
Iridocyclitis	5 (13.2)
Vision blurred	3 (7.9)
Visual acuity reduced	2 (5.3)
Glare	2 (5.3)
Periorbital swelling	2 (5.3)
Myopia	1 (2.6)
Halo vision	1 (2.6)
Visual impairment	1 (2.6)
Dry eye	1 (2.6)
Ocular discomfort	1 (2.6)
Diplopia	1 (2.6)
Clavicle fracture	1 (2.6)
Periarthritis	1 (2.6)
Migraine	1 (2.6)
Renal cyst	1 (2.6)
Dermatitis contact	1 (2.6)

## DISCUSSION

This multicenter prospective clinical trial confirmed the effectiveness and safety of first-line treatment with once-daily omidenepag isopropyl 0.002% ophthalmic solution in patients with newly diagnosed POAG who had not previously received topical ophthalmic treatment.

The main finding was the significant reduction by 16% (−2.6 mm Hg) at week 12 in mean IOP from the baseline mean of 16.19 mm Hg (*P*<0.0001); 43% of patients recorded a decrease in IOP of more than 20%. Omidenepag isopropyl 0.002% ophthalmic solution had a rapid IOP-lowering effect as the percentage reduction from baseline was 15% at week 4. Omidenepag isopropyl 0.002% ophthalmic solution was equally effective in the NTG subgroup who also had a significant 16% reduction at week 12 in mean IOP from a baseline mean of 15.79 mm Hg. Given that the challenge of lowering IOP becomes greater with lower baseline IOP,^19^ the effectiveness of omidenepag isopropyl in the NTG subgroup is a particularly relevant finding for clinical practice. The IOP-lowering effect of omidenepag isopropyl 0.002% ophthalmic solution observed in daily clinical practice aligns with results of phase 3 studies, which reported reductions of 15%–35%.^12–17^

Relative to the phase 3 studies,^12–17^ our patient population was younger (50 vs. >60 y) and mean baseline IOP was lower (16.2 vs. 23–24 mm Hg), likely due to differences in patient profiles (newly-diagnosed vs. previously-treated/ low-responder or nonresponder patients) and minimum baseline IOP (10 vs. ≥22 mm Hg). Although we cannot explain the difference in sex distribution between our study and the phase 3 studies (67 vs. ∼50% male), we consider it unlikely to have had any impact on the results. The characteristics of our patient population in terms of age, baseline IOP, and proportion with NTG align closely with the profile of patients with newly diagnosed POAG and NTG in Korea. A retrospective review of 5530 patients attended at 20 major hospital and ophthalmology outpatient clinics in major cities of Korea found that the most frequently observed glaucoma subtypes were NTG (33.0%) and POAG (28.4%). Mean age of patients with these subtypes were 51.4 and 52.7 years, respectively, and mean IOP was 16.3 and 22.2 mm Hg, respectively.^20^

A comparison of outcomes in our cohort with those recorded in the treatment-naive groups of 2 real-world Japanese studies of omidenepag isopropyl 0.002% ophthalmic solution reveals marked similarities. Both Japanese studies presented outcomes separately for 3 patient subgroups: naive monotherapy, switching monotherapy, and concomitant therapy.^21,22^ At 12-month interim analysis of a prospective, multicenter postmarketing study, 902 patients comprised the naive monotherapy group, 235 (26%) with POAG and 621 (69%) with NTG. Across this group, mean IOP was reduced by −2.7±2.6 mm Hg at 12 months from the baseline mean of 17.0±3.8 mm Hg (*P*<0.05), equivalent to a 15% reduction.^21^ In a retrospective medical chart review study conducted at 11 eye clinics in Japan, 283 (83.0%) of 341 patients in the naive monotherapy group were diagnosed with POAG/NTG. Mean IOP decreased from 16.6±4.2 mm Hg at baseline to 14.0±3.3 mm Hg at 12 weeks for a mean change of −2.5±2.9 mm Hg (*P*<0.0001), also representing a 15% reduction.^22^

In our cohort, the number of cases of conjunctival injection reported with omidenepag isopropyl 0.002% ophthalmic solution increased from baseline to week 4, then decreased to week 12 suggesting that patients became accustomed or adapted to the medication with continued use. Although some phase 3 studies of omidenepag isopropyl 0.002% ophthalmic solution have reported sporadic cases of macular edema, cystoid macular edema, and corneal thickening,^14,16^ in our cohort, either no cases occurred or numerical changes in ocular indicators were minimal during 12 weeks’ observation.

Cases of DUES reported in 2 patients at 4 and 12 weeks were assessed as mild, and the patients continue to be followed. DUES may appear more pronounced in Asians due to their characteristic eyelid anatomy.^23^ Photography is currently the only tool available to measure DUES objectively and may be used infrequently outside the clinical trial setting. To facilitate the evaluation of PAPS in real-world clinical environments, validated patient-reported outcome measures need to be developed. Importantly, 52-week safety results from phase III studies conducted in Japan^16^ and the United States^17^ lend support to a report indicating little to no induction of PAPS with omidenepag isopropyl compared with tafluprost in patients with POAG or ocular hypertension.^24^

Overall, omidenepag isopropyl 0.002% ophthalmic solution was well tolerated. Twenty-three patients experienced AEs, of which almost all were reported as mild. Consistent with the safety profile of omidenepag isopropyl 0.002% ophthalmic solution during its clinical development,^25^ the most common AE in our cohort was hyperemia. Likewise, ocular hyperemia was the most frequent adverse drug reaction reported in real-world Japanese studies of omidenepag isopropyl 0.002% ophthalmic solution, with incidences of 3.5% at 12 months and 9.7% at 12 weeks in the naive monotherapy group of the respective studies.^21,22^ As all 5 cases of iridocyclitis were mild and no action was required, we were not overly concerned. In fact, we regard the finding as a study artifact that arose from using the term “iridocyclitis” to categorize cases of anterior uveitis. By comparison, iridocyclitis was not among the adverse drug reactions reported in either of the real-world Japanese studies of omidenepag isopropyl 0.002% ophthalmic solution.^21,22^

General limitations with observational studies include the absence of a control group and the potential for bias. A specific limitation of our study is that the number of patients analyzed did not meet the target sample size. Study strengths are the prospective, multicenter design, and the inclusion of treatment-naïve patients with newly diagnosed POAG, which allowed us to evaluate the suitability of omidenepag isopropyl 0.002% ophthalmic solution for first-line use.

## CONCLUSIONS

This prospective phase IV study is the first to investigate omidenepag isopropyl 0.002% ophthalmic solution in newly diagnosed POAG and NTG in Korea. The results corroborate its effectiveness and safety as demonstrated during clinical development and in large-scale observational studies conducted in real-world clinical settings in Japan. Omidenepag isopropyl effectively lowers IOP and has a favorable safety profile. The study is particularly meaningful clinically as it describes the first-line use of omidenepag isopropyl 0.002% ophthalmic solution in treatment-naive patients in Korea and demonstrates a significant IOP-lowering effect also in POAG patients with NTG.
